# The hedgehog and Wnt/β-catenin system machinery mediate myofibroblast differentiation of LR-MSCs in pulmonary fibrogenesis

**DOI:** 10.1038/s41419-018-0692-9

**Published:** 2018-05-29

**Authors:** Xiang Chen, Chaowen Shi, Honghui Cao, Ling Chen, Jiwei Hou, Zou Xiang, Kebin Hu, Xiaodong Han

**Affiliations:** 1 Immunology and Reproduction Biology Laboratory & State Key Laboratory of Analytical Chemistry for Life Science, Medical School, Nanjing University, Nanjing, Jiangsu 210093 China; 2 Jiangsu Key Laboratory of Molecular Medicine, Nanjing, 210093 China; 3 Department of Health Technology and Informatics, Faculty of Health and Social Sciences, The Hong Kong Polytechnic University, Hung Hom, Kowloon, Hong Kong China; 4 Department of Medicine, Division of Nephrology, Penn State University College of Medicine, Hershey, PA 17033 USA

## Abstract

Idiopathic pulmonary fibrosis (IPF) is a chronic, progressive and fatal lung disease that is characterized by enhanced changes in stem cell differentiation and fibroblast proliferation. Resident mesenchymal stem cells (LR-MSCs) can undergo phenotype conversion to myofibroblasts to augment extracellular matrix production, impairing function and contributing to pulmonary fibrosis. Hedgehog and Wnt signaling are developmental signal cascades that play an essential role in regulating embryogenesis and tissue homeostasis. Recently, it has been reported that both hedgehog and Wnt signaling play important roles in pulmonary fibrogenesis. Thus, the identification of specific target regulators may yield new strategy for pulmonary fibrosis therapies. In our work, we demonstrated the critical role of Gli1, Wnt7b, Wnt10a and Fzd10 in the process of pulmonary fibrogenesis in vitro and in vivo. Gli1 was induced in LR-MSCs following TGF-β1 treatment and fibrotic lung tissues. Inhibition of Gli1 suppressed myofibroblast differentiation of LR-MSCs and pulmonary fibrosis, and decreased the expression of Wnt7b, Wnt10a and β-catenin. Gli1 bound to and increased promoter activity of the Wnt7b and Wnt10a genes, and Wnt7b and Wnt10a were critical activators of Wnt/β-catenin signaling. It was noteworthy that Fzd10 knockdown reduced Wnt7b and Wnt10a-induced activation of Wnt/β-catenin signaling, which imply that Wnt7b and Wnt10a may be the ligands for Fzd10. Moreover, siRNA-mediated inhibition of Fzd10 prevented TGF-β1-induced myofibroblast differentiation of LR-MSCs in vitro and impaired bleomycin-induced pulmonary fibrosis. We conclude that hedgehog and Wnt/β-catenin signaling play a critical role in promoting myofibroblast differentiation of LR-MSCs and development of pulmonary fibrosis. These findings elucidate a therapeutic approach to attenuate pulmonary fibrosis through targeted inhibition of Gli1 or Fzd10.

## Introduction

Idiopathic pulmonary fibrosis (IPF) is a chronic, progressive and lethal disease of uncertain etiology^1^. It has been reported that IPF may be a result of the migration, proliferation and activation of mesenchymal cells provoked by the aberrant activation of alveolar epithelial cells after injury. In this process, the activated mesenchymal cells would lead to the formation of fibroblastic/myofibroblastic foci and exaggerated accumulation of extracellular matrix (ECM), resulting in irreversible destruction of the lung parenchyma^2–4^. The presence of fibroblastic/myofibroblastic foci is an important prognostic factor, as their numbers have been correlated with survival in IPF^5,6^. However, the molecular mechanisms of IPF remain poorly understood.

The Wnt/β-catenin signaling pathway was found dysregulated in the lung tissues from patients with IPF^7,8^. Wnt/β-catenin signaling has a profound effect on developmental processes during embryogenesis and plays a key role for tissue homeostasis in adults^9,10^. Wnt proteins are a family of secreted molecules that transmit signal by interacting with Frizzled receptors and low-density lipoprotein receptor-related protein co-receptors (LRP5/6)^11,12^, which results in β-catenin translocating to nucleus and binding with T-cell factor/lymphoid enhancer-binding factor (Tcf/Lef) to promote the transcription of Wnt target genes^13^. Wnt proteins have been found to be expressed at low levels in the normal lung and may be associated with epithelial turnover^8,14^. So far, aberrant activation of Wnt/β-catenin signaling that is mediated by altered expressions of Wnt proteins has been reported to be implicated in various diseases, including renal, pulmonary and liver fibrosis^15–17^. Emerging evidence showed that mesenchymal stem cells (MSCs) could influence the development of inflammation and injury model systems^18,19^. MSCs could regulate fibroblast growth and collagen production through expressing paracrine factors, like Wnt proteins^20^. However, the molecular mechanism of dysregulated Wnt proteins in pulmonary fibrogenesis remains enigmatic.

Previous research has reported that hedgehog signaling could induce the expression of Wnts through glioma-associated oncogene homolog (GLI) family members which could bind with the promoter area of Wnt genes^21–23^. In the development of IPF, hedgehog signaling is robustly activated, resulting in increased proliferation of fibroblasts and ECM synthesis^24^. Hedgehog signal transduction is mediated by two transmembrane proteins: the hedgehog-binding receptor Patched-1 (PTC) and Smoothened (SMO), and finally resulting in the activation of GLI transcription factor family which induce the expression of GLI downstream targets^25–27^. In the absence of hedgehog ligand, GLI1 is not expressed, whereas GLI2 and GLI3 are processed into, respectively, a weak and a strong transcriptional repressor^27^. However, the relationship between aberrant expressed GLI and Wnt remains poorly illuminated.

In the present study, we verified that Wnt signaling and hedgehog signaling were both activated in pulmonary fibrogenesis. Thus, we comprehensively characterized Wnt, Fzd and Gli gene expression patterns of myofibroblast-differentiated lung-resident mesenchymal stem cells (LR-MSCs) and fibrotic lung tissues. We reported extremely increased expression of Wnt7b/10a, Fzd9/10 and Gli1 in the differentiation of LR-MSCs and lung fibrosis. In particular, we demonstrated that Wnt7b and Wnt10a could bind with Fzd10 to activate Wnt/β-catenin signaling. Targeted inhibition of Fzd10 could evidently suppress the development of pulmonary fibrosis. In addition, blocking hedgehog signaling by Gli1 inhibitor could also impair pulmonary fibrosis through defecting the transcription of Wnt7b/10a which were the target genes of Gli1. Thus, our study identified Wnt/β-catenin signaling and hedgehog signaling crosstalk in pulmonary fibrosis in vitro and in vivo, and highlighted Gli1 and Fzd10 as a potential therapeutic target in pulmonary fibrosis.

## Results

### LR-MSCs could differentiate into myofibroblasts in vivo

**Fig. 1:**
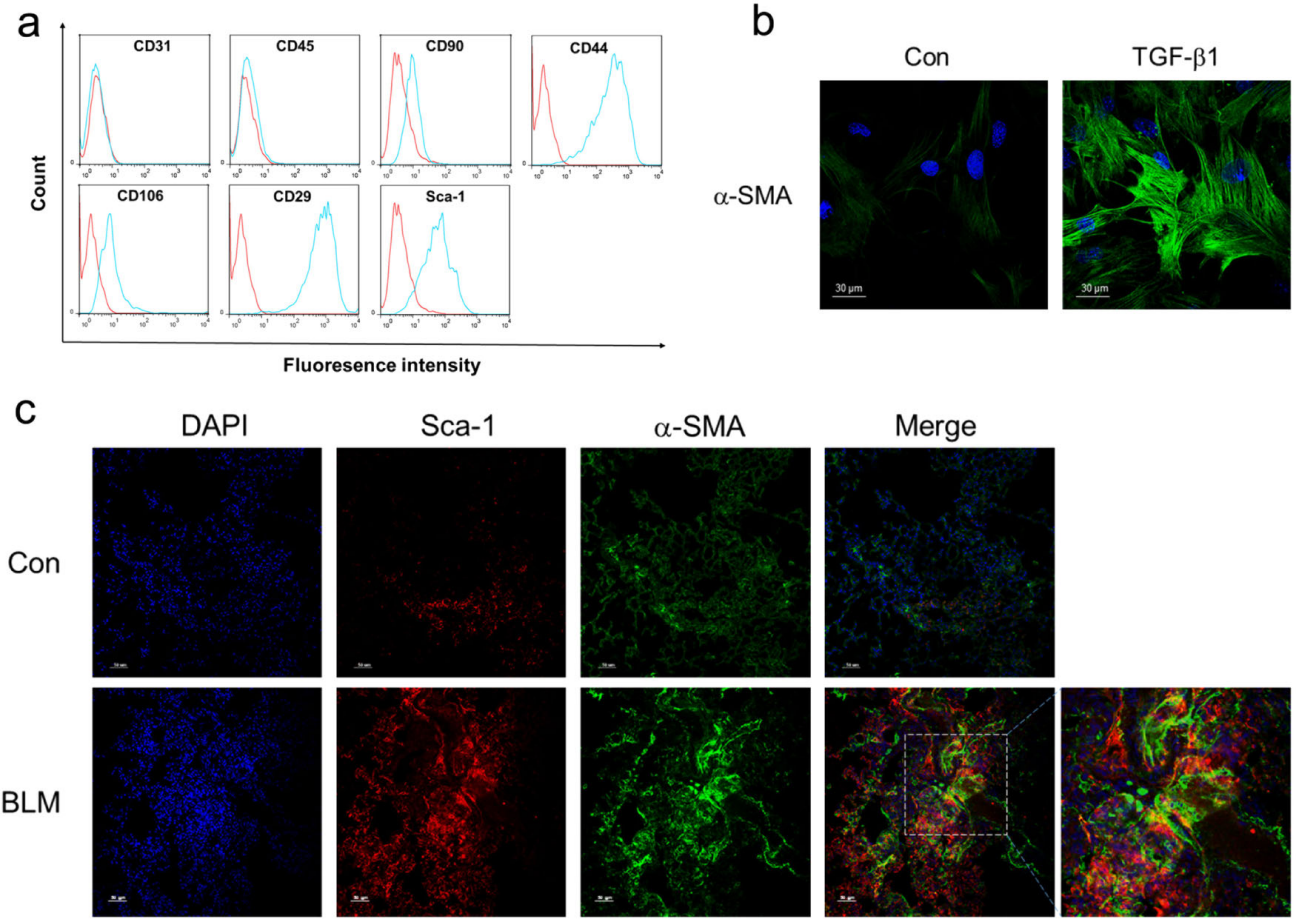
Lung-resident mesenchymal stem cells (LR-MSCs) expand and become myofibroblasts in bleomycin (BLM)-induce pulmonary fibrosis. **a** The expression of Sca-1, CD29, CD31, CD44, CD45, CD90 and CD106 was measured by flow cytometry. Red lines indicate isotype control staining and blue lines specific antibody staining. **b** The expression of α-smooth muscle actin (α-SMA) in LR-MSCs treated with TGF-β1 was determined by immunofluorescence staining. **c** The levels of α-SMA and Sca-1 in the lung tissues of the BLM-induced pulmonary fibrosis model were examined by immunofluorescence staining

The primary LR-MSCs from mouse lung tissues were isolated by Magnetic-activated cell sorting (MACS) as previously reported. The purity of LR-MSCs was confirmed by the expression of CD90, CD44, CD106, CD29 and Sca-1, but not CD45 and CD31 (Fig. 1a). In our previous study, we found that LR-MSCs could differentiate into myofibroblasts in the treatment of transforming growth factor-β1 (TGF-β1; Fig. 1b)^28^. To determine whether LR-MSCs have a fibrosis-inducing role in pulmonary fibrosis, we performed fate-tracing experiment in a murine model of bleomycin (BLM)-induced pulmonary fibrosis. To trace the fate of LR-MSCs, lung sections were double stained with Sca-1 (a marker of LR-MSCs) and α-smooth muscle actin (α-SMA; a marker of myofibroblasts). Confocal imaging demonstrated that LR-MSCs undergo tremendous expansion, and acquire expression of α-SMA, indicating myofibroblast differentiation of LR-MSCs in pulmonary fibrogenesis (Fig. 1c).

### Wnt genes were differentially expressed in myofibroblast-differentiated LR-MSCs and fibrotic lungs

**Fig. 2:**
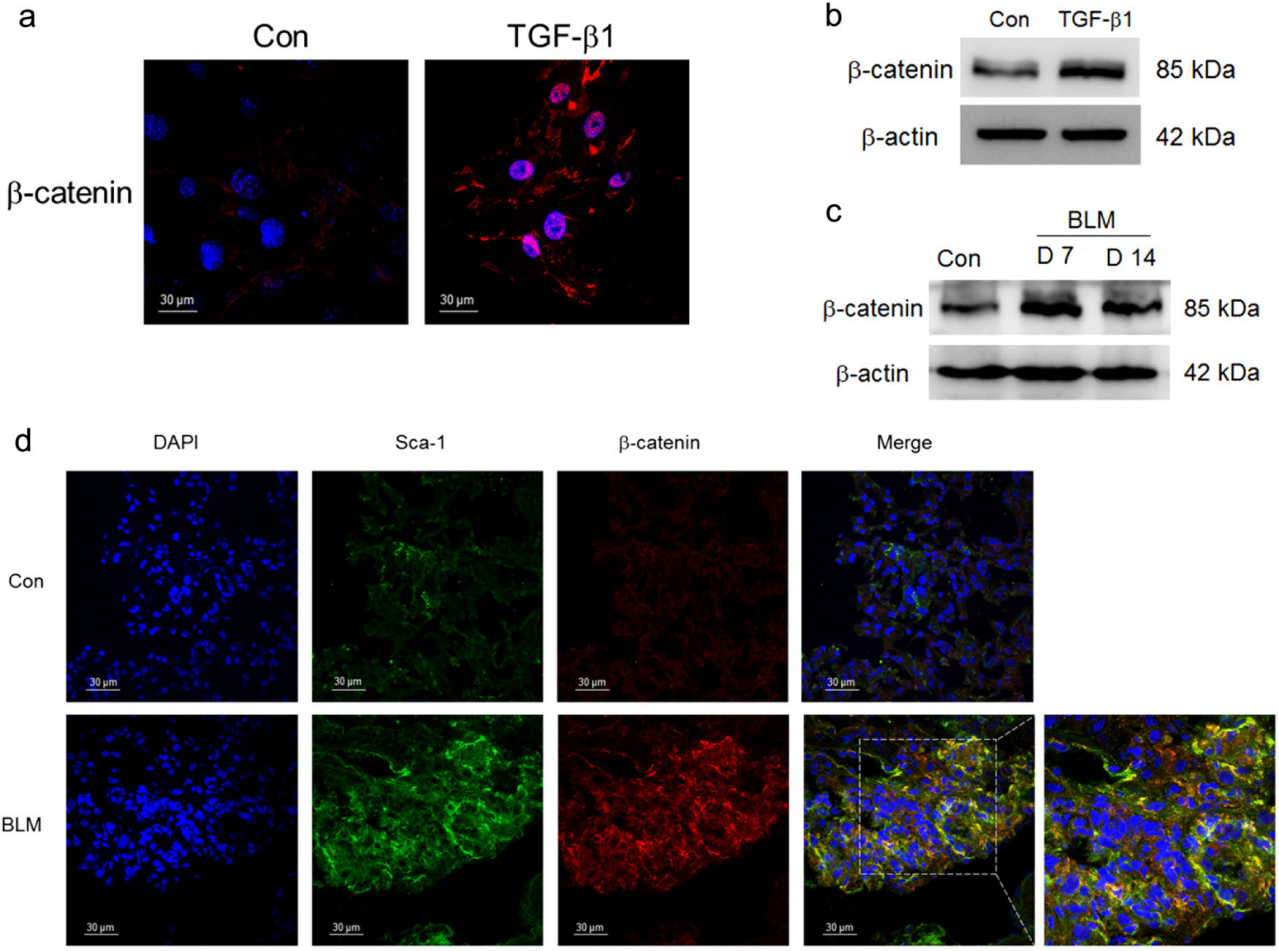
Wnt/β-catenin signaling was aberrantly activated in myofibroblast differentiation of lung-resident mesenchymal stem cells (LR-MSCs) in vitro and in vivo. **a**, **b** The expression of β-catenin in LR-MSCs with the treatment of TGF-β1 was measured by immunofluorescence staining (**a**) and western blot (**b**). **c**, **d** The lung tissues of the bleomycin (BLM)-induced pulmonary fibrosis model were double stained with β-catenin and Sca-1 by immunofluorescence staining (**d**) and the expression of β-catenin was further determined by western blot (**c**). Data represented three independent experiments

To investigate the activity of Wnt/β-catenin signaling, we first measured the expression of β-catenin in LR-MSCs treated with TGF-β1. We found that β-catenin was extensively expressed in the myofibroblast differentiation of LR-MSCs in vitro, which indicate the activation of Wnt/β-catenin signaling (Fig. 2a, b). The results were additionally confirmed in the lung tissues derived from pulmonary fibrosis mouse model (Fig. 2c). Wnt/β-catenin signaling was profoundly activated in LR-MSCs (Sca-1^+^) in the development of pulmonary fibrosis (Fig. 2d).

**Fig. 3:**
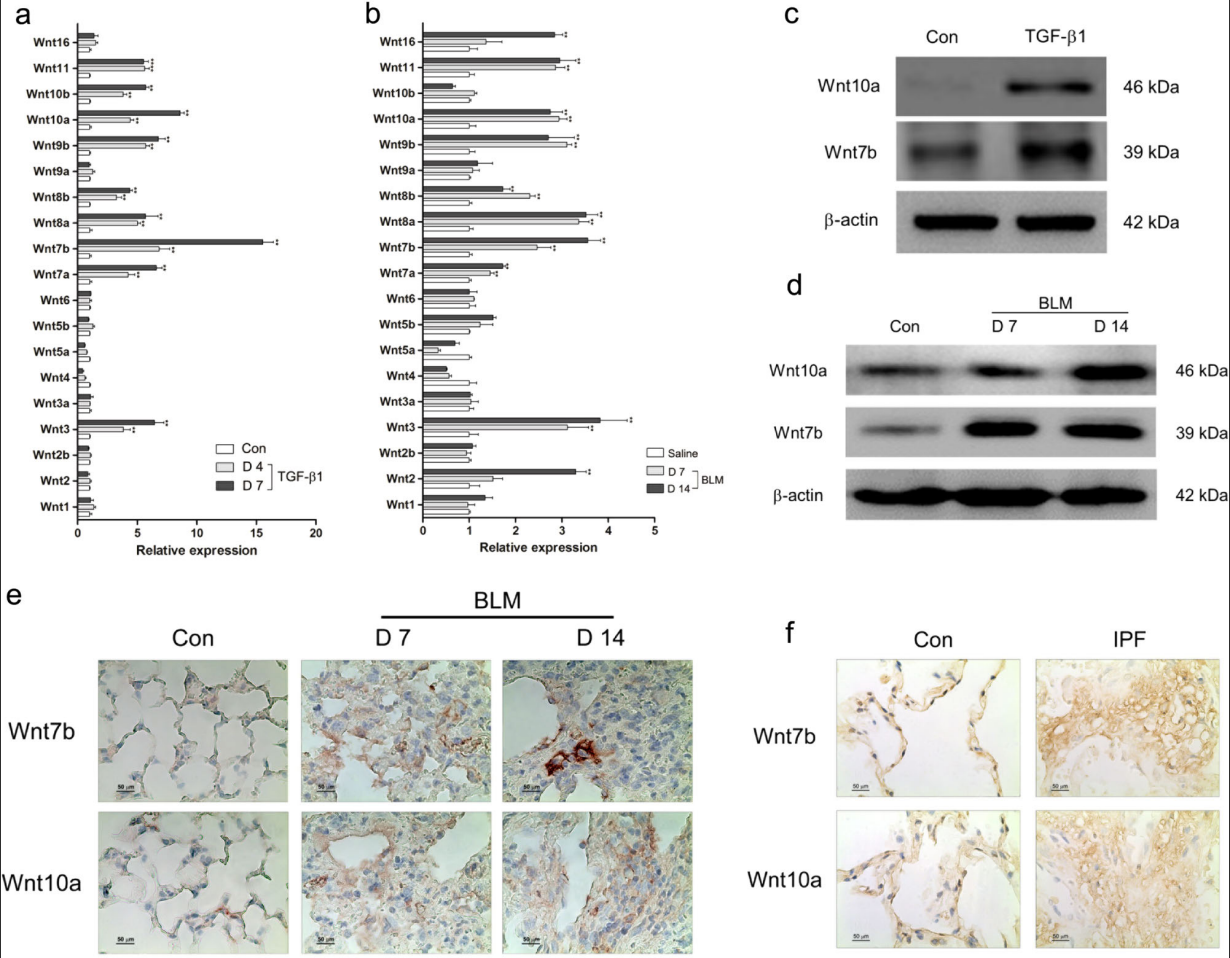
Wnt genes were differentially expressed in myofibroblast differentiation of lung-resident mesenchymal stem cells (LR-MSCs) and fibrotic lung tissues. **a**, **b** Quantitative PCR analysis showed the relative mRNA levels of Wnt genes in TGF-β1-treated LR-MSCs (**a**) and bleomycin (BLM)-induced fibrotic lung tissue (**b**). **c** Levels of Wnt7b and Wnt10a in LR-MSCs treated with TGF-β1 were measured by western blot. **d**, **e** The expression of Wnt7b and Wnt10a in BLM-induced mouse fibrotic lung tissues was determined by western blot (**d**) and immunohistochemistry (**e**). **f** Immunodetection showed Wnt7b and Wnt10a in human normal (Con) lung tissues (left panel) and IPF lung tissues (right panel). Data represented three independent experiments

In order to confirm the roles of Wnt family members that played in pulmonary fibrogenesis, we first performed a systematic analysis of the messenger RNA (mRNA) level of 19 Wnt genes in both fibrotic mouse lung tissues and myofibroblast-differentiated LR-MSCs. We found that 8 Wnt genes (Wnt3, Wnt7a, Wnt7b, Wnt8a, Wnt8b, Wnt9b, Wnt10a, Wnt11) were upregulated both in vitro and in vivo (Fig. 3a, b). Among the 8 Wnt genes, Wnt7b and Wnt10a were especially overexpressed in vitro or in vivo. Moreover, it was ever reported that Wnt7b and Wnt10a were severely expressed in the lung tissues from IPF patients^15,29^. As shown in Fig. 3c–e, the protein levels of Wnt7b and Wnt10a were greatly increased in both myofibroblast-differentiated LR-MSCs and fibrotic lung tissues. We also confirm these findings in the lung tissues from IPF patients (Fig. 3f).

### Hedgehog signaling was aberrantly activated in pulmonary fibrogenesis

**Fig. 4:**
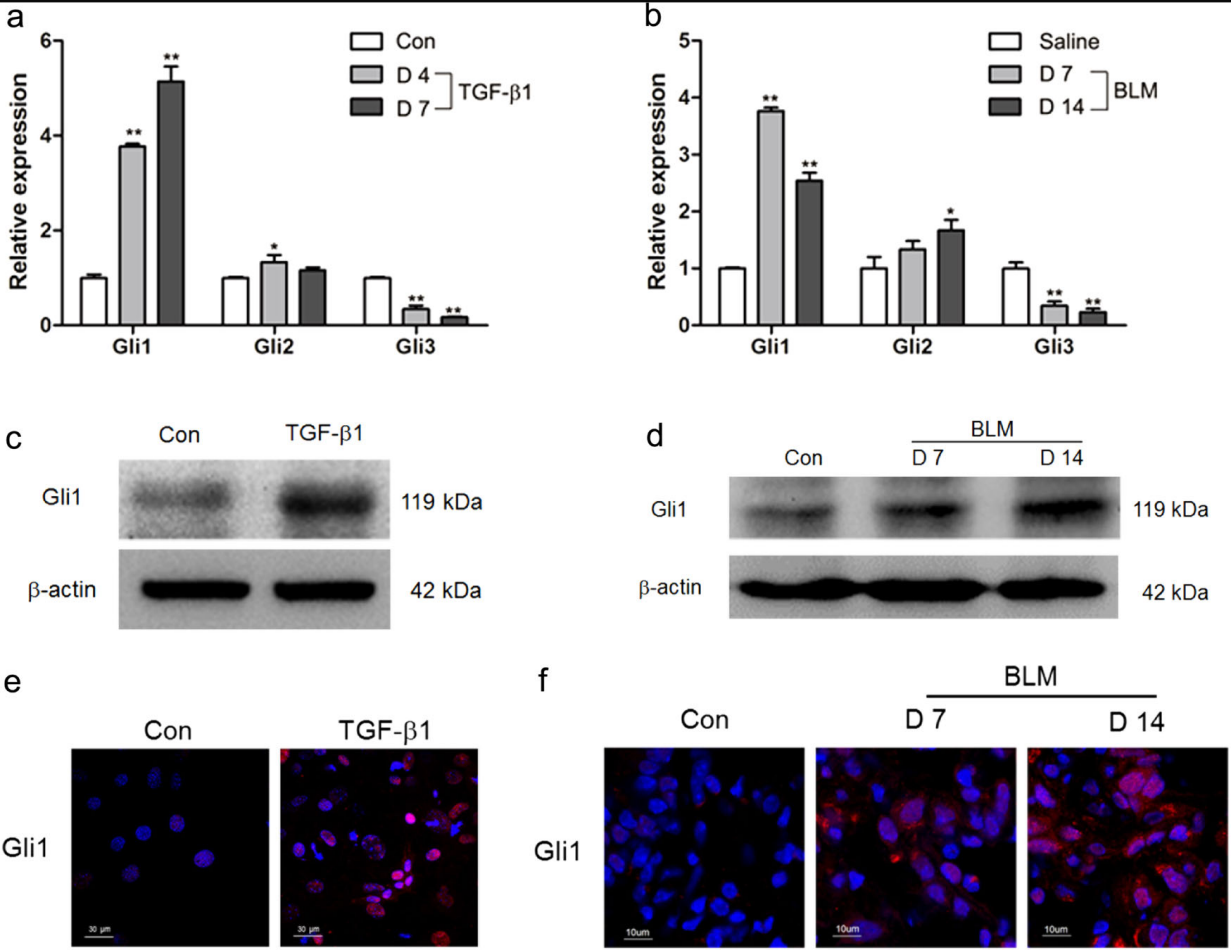
Hedgehog signaling was aberrantly activated in myofibroblast differentiation of lung-resident mesenchymal stem cells (LR-MSCs) and fibrotic lung tissues. **a**, **b** The expressions of Gli1, Gli2 and Gli3 in TGF-β1-treated LR-MSCs (**a**) and bleomycine (BLM)-induced mouse fibrotic lung tissues (**b**) were analyzed by quantitative PCR. * *P* < 0.05 vs. Con or Saline, ** *P* < 0.01 vs. Con or Saline. **c**–**f** The level of Gli1 in TGF-β1-treated LR-MSCs (**c**, **e**) and bleomycine (BLM)-induced mouse fibrotic lung tissues (**d**, **f**) was determined by western blot (**c**, **d**) and immunofluorescence staining (**e**, **f**)

It was previously observed that hedgehog signaling cascade cross-talks with Wnt signaling to regulate the balance of stem cells and adult tissue homeostasis^30,31^. To investigate the functional role of hedgehog signaling in pulmonary fibrosis, the mRNA levels of Gli were measured in TGF-β1-treated LR-MSCs and fibrotic lung tissues. The results showed that Gli1 was greatly elevated both in vitro and in vivo (Fig. 4a–d). In addition, the results showed that Gli1 was dramatically expressed and translocates to nucleus in myofibroblast-differentiated LR-MSCs and lung tissues derived from pulmonary fibrosis mouse models (Fig. 4e, f).

### GANT58 could suppress pulmonary fibrosis through inhibiting Gli1

**Fig. 5:**
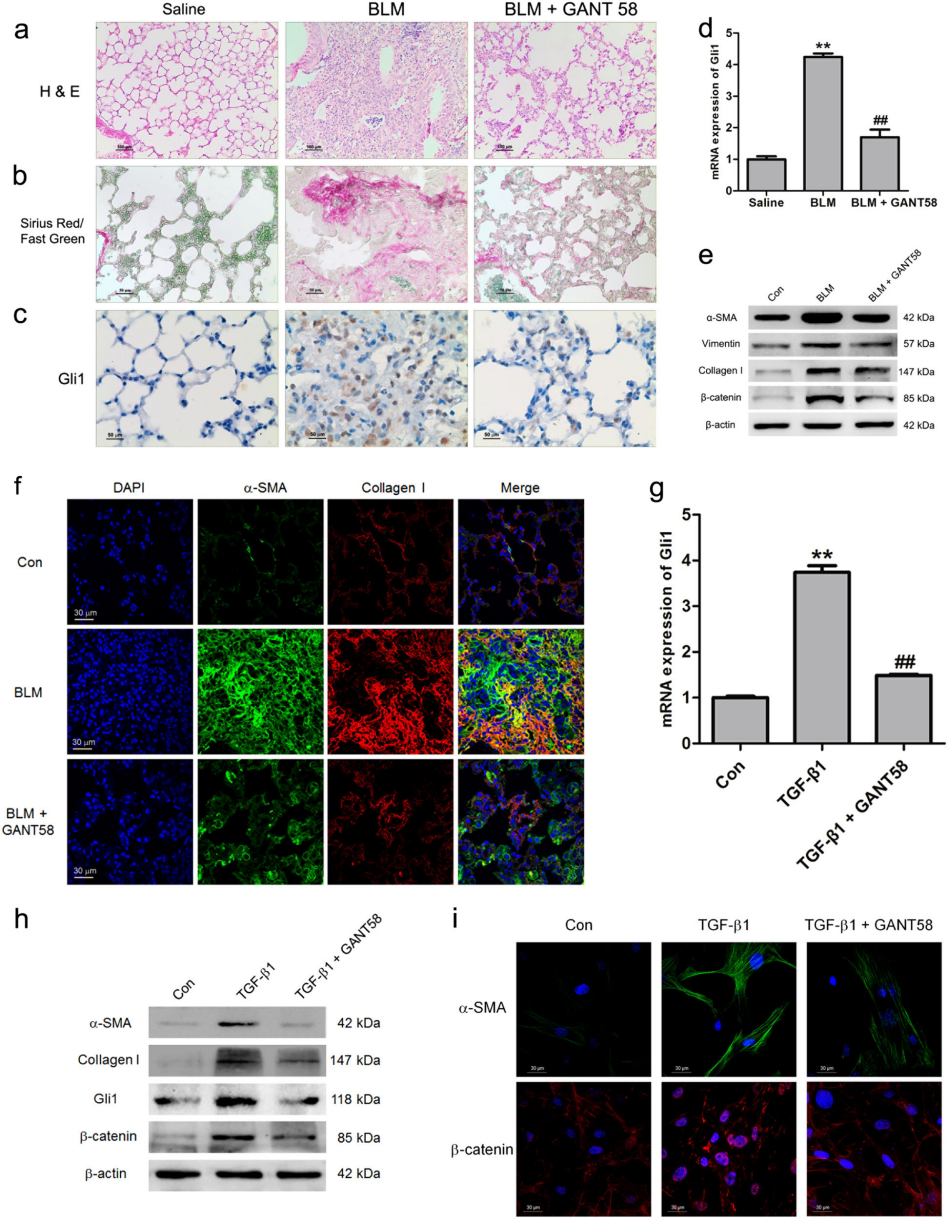
GANT58 attenuated pulmonary fibrosis through Gli1 inhibition in vitro and in vivo. Mice (*n* = 10 in each group) were intraperitoneally injected with vehicle (10% DMSO/PBS) or 25 mg/kg GANT58 every other day as indicated 7 days after the administration of BLM. **a** Pulmonary fibrosis was determined by hematoxylin–eosin (H&E) staining. **b** Collagen was revealed by Sirius Red/Fast Green staining. **c**, **d** The expression of Gli1 in lung tissues was measured by immunohistochemistry (**c**) and quantitative PCR (**d**). Representative micrographs of histology are shown; **P < 0.01 vs. saline, ^##^*P* < 0.01 vs. BLM. **e** The levels of α-SMA, vimentin, Collagen I and β-catenin were examined by western blot. **f** The expression of α-SMA and Collagen I was determined by immunofluorescence staining. Lung-resident mesenchymal stem cells (LR-MSCs) were cultured with TGF-β1 for 7 days, along with or without the treatment of GANT58 (10 μM). **g** The mRNA level of Gli1 was measured by quantitative PCR. **P < 0.01 vs. Con, ^##^*P* < 0.01 vs. TGF-β1. **h** The protein levels of α-SMA, Collagen I, Gli1 and β-catenin were examined by western blot. **i** The expression of α-SMA and β-catenin was further determined by immunofluorescence staining

We next investigated whether inhibition of hedgehog signaling could block the development of pulmonary fibrosis. GANT58, a chemical molecular inhibitor of Gli1, was chosen to suppress the hedgehog signaling. Compared with BLM-treated mice, GANT58 evidently reduced the extent of lung lesions on day 14 (Fig. 5a) and suppressed collagen deposition as assessed with Sirius red/fast green collagen staining (Fig. 5b). GANT58 decreased the content of Gli1 and the percentage of Gli1-positive cells (Fig. 5c). Moreover, Gli1, as a target gene of hedgehog signaling, was extremely suppressed in the treatment of GANT58 (Fig. 5d), indicating the inhibition of hedgehog signaling in vivo. Immunolabeling also showed that GANT58 profoundly attenuated the expression of fibrotic markers α-SMA, Collagen I and Vimentin (Fig. 5e, f) and inhibited the activation of Wnt/β-catenin signaling by impairing the expression of β-catenin (Fig. 5e). These results demonstrate that inhibition of Gli1 with GANT58 could protect mice from BLM-induced pulmonary fibrosis.

Having demonstrated that the inhibition of hedgehog signaling could prevent the development of BLM-induced pulmonary fibrosis in vivo, we next used GANT58 to investigate whether inhibiting hedgehog signaling was sufficient to suppress TGF-β1-induced myofibroblast differentiation of LR-MSCs in vitro. As shown in Fig. 5g, h, GANT58 could effectively suppress the expression of Gli1 which was extremely expressed in myofibroblast-differentiated LR-MSCs. When LR-MSCs were stimulated with TGF-β1 in the presence of GANT58, the myofibroblast differentiation of LR-MSCs was evidently suppressed as shown by impaired expression of α-SMA and Collagen I (Fig. 5h, i). In addition, inhibition of Gli1 could also block TGF-β1-induced activation of canonical Wnt signaling through impairing nuclear accumulation of β-catenin (Fig. 5h, i), which was consistent with the results found in vivo.

### Wnt genes were regulated by Gli1 in the myofibroblast differentiation of LR-MSCs and fibrotic lungs

**Fig. 6:**
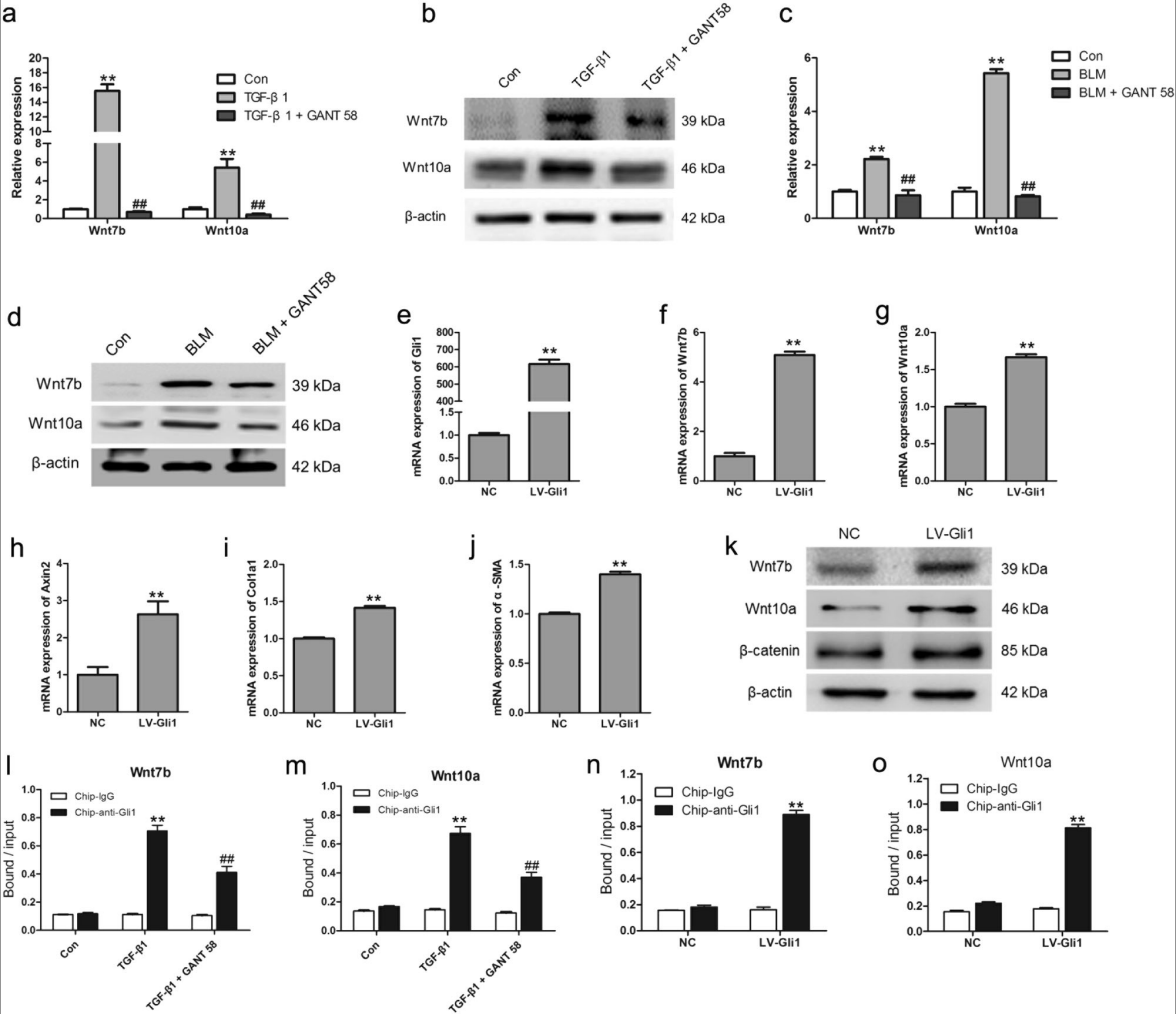
Wnt7b and Wnt10a were regulated by cellular DNA binding of Gli1. Lung-resident mesenchymal stem cells (LR-MSCs) were treated as in Fig. 6. The mRNA levels (**a**) and protein levels (**b**) of Wnt7b and Wnt10a were measured by quantitative PCR and western blot. Mice were treated as in Fig. 5. The mRNA levels (**c**) and protein levels (**d**) of Wnt7b and Wnt10a in lung tissues were measured by quantitative PCR and western blot. **e**–**j** The mRNA level of Gli1, Wnt7b, Wnt10a, Col1a1, α-SMA and Axin2 in LR-MSCs transfected with LV-Gli1 or negative control (NC) were measured by quantitative PCR. **k** The protein levels of Wnt7b, Wnt10a and β-catenin were determined by western blot. **l**, **m** ChIP analysis for Gli1 binding to Wnt7b and Wnt10a in LR-MSCs treated with TGF-β1, along with or without GANT58. **n**, **o** ChIP analysis for Gli1 binding to Wnt7b and Wnt10a in LR-MSCs transfected with LV-Gli1 or NC. Enrichment of promoter regions was normalized by input. **P < 0.01 vs. Con, ^##^*P* < 0.01 vs. TGF-β1 or BLM

To investigate the regulation mechanism between Gli1 and β-catenin, we further measured the levels of Wnt7b and Wnt10a after inhibiting Gli1 by GANT58 in vitro and in vivo. We found that Wnt7b and Wnt10a, which were extensively expressed in pulmonary fibrogenesis, were profoundly suppressed by GANT58 both in vitro and in vivo (Fig. 6a–d). In addition, when LR-MSCs were transfected with Gli1 vector, the expressions of Wnt7b and Wnt10a were elevated, accompanied with the overexpression of β-catenin (Fig. 6e–g, k). Overexpressing Gli1 could also enhance the expression of Axin2 which was the target gene of Wnt/β-catenin signaling (Fig. 6h), and the mRNA level of α-SMA and Col1a1 (Fig. 6i, j). We next investigated whether TGF-β1 facilitated the recruitment of Gli1 to Wnt7b and Wnt10a promoters. Chromatin immunoprecipitation (ChIP) assay with pull-down using anti-Gli1 Ab revealed increased occupancy by Gli1 at the regions of the Wnt7b and Wnt10a promoter in response to TGF-β1, which was inhibited by GANT58 (Fig. 6l, m). Overexpression of Gli1 in LR-MSCs increased anti-Gli1-mediated enrichment of Wnt7b and Wnt10a promoter regions (Fig. 6n, o). These results strongly indicated that Gli1 may induce the activation of Wnt/β-catenin signaling by promoting the expression of Wnt7b and Wnt10a.

### Wnt7b/10a-promoted LR-MSCs differentiate into myofibroblasts through activating Wnt/β-catenin signaling

**Fig. 7:**
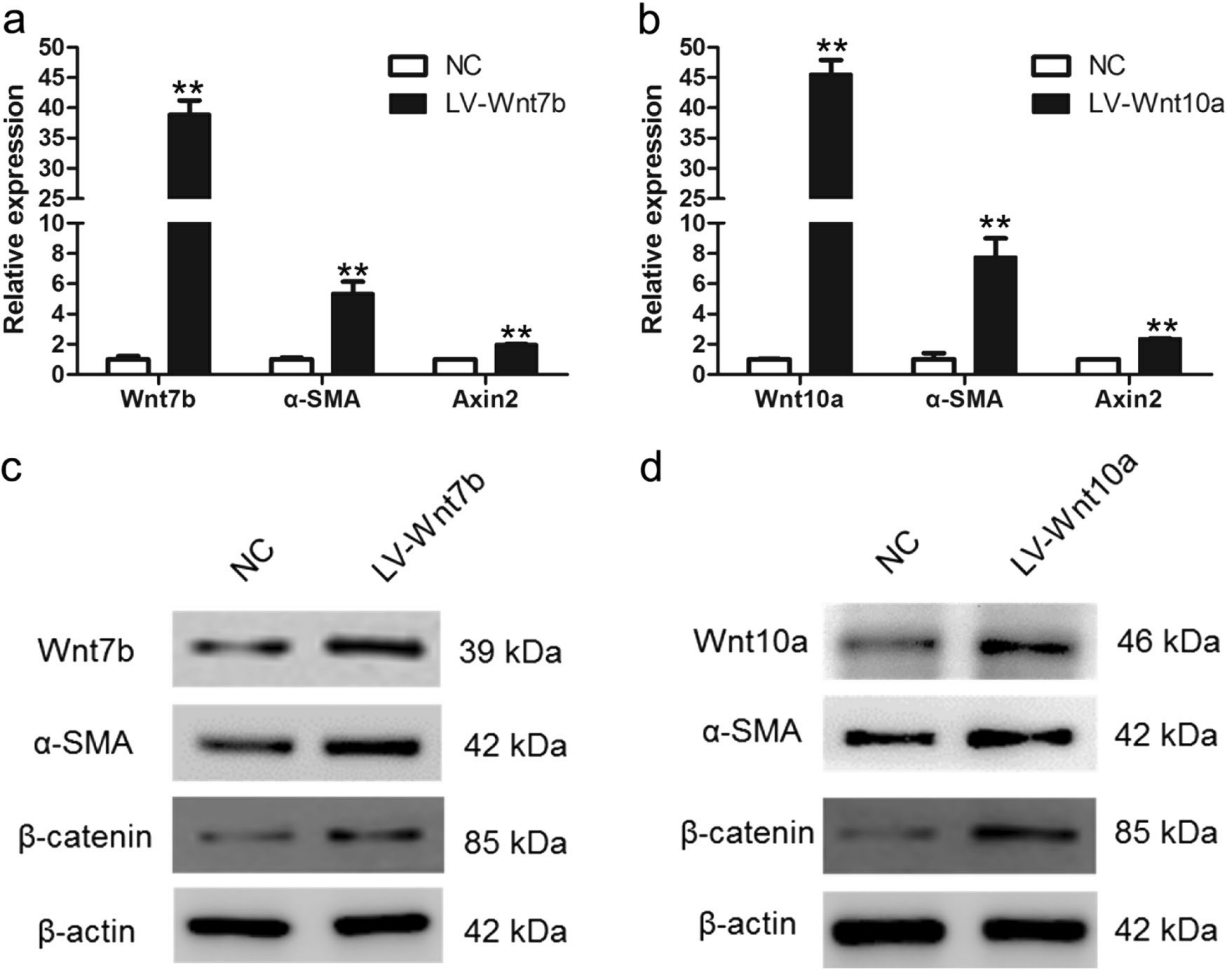
Wnt7b and Wnt10a promoted myofibroblast differentiation of lung-resident mesenchymal stem cells (LR-MSCs) via activating canonical Wnt signaling. LR-MSCs were transfected with LV-Wnt7b or LV-Wnt10a or negative control (NC). **a**, **b** The expressions of Wnt7b, Wnt10a, α-SMA and Axin2 were examined by quantitative PCR. ** *P* < 0.05 vs. NC. **c**, **d** The protein levels of Wnt7b, Wnt10a, α-SMA and β-catenin were examined by western blot

In order to determine whether Wnt7b/10a is responsible for the differentiation of LR-MSCs, or simply a consequence of fibrosis, we examined the mRNA levels of α-SMA and Axin2 in LR-MSCs which were transfected with LV-Wnt7b or LV-Wnt10a. Overexpression of Wnt7b or Wnt10a could increase the expression of α-SMA and Axin2 (Fig. 7a, b). Similarly, the protein levels of α-SMA and β-catenin were dramatically elevated after transfecting Wnt7b or Wnt10a vector (Fig. 7c, d). These date indicated that Wnt7b and Wnt10a could promote myofibroblast differentiation of LR-MSCs through activating Wnt/β-catenin signaling.

### Wnt receptors were differentially expressed in myofibroblast-differentiated LR-MSCs and fibrotic lungs

**Fig. 8:**
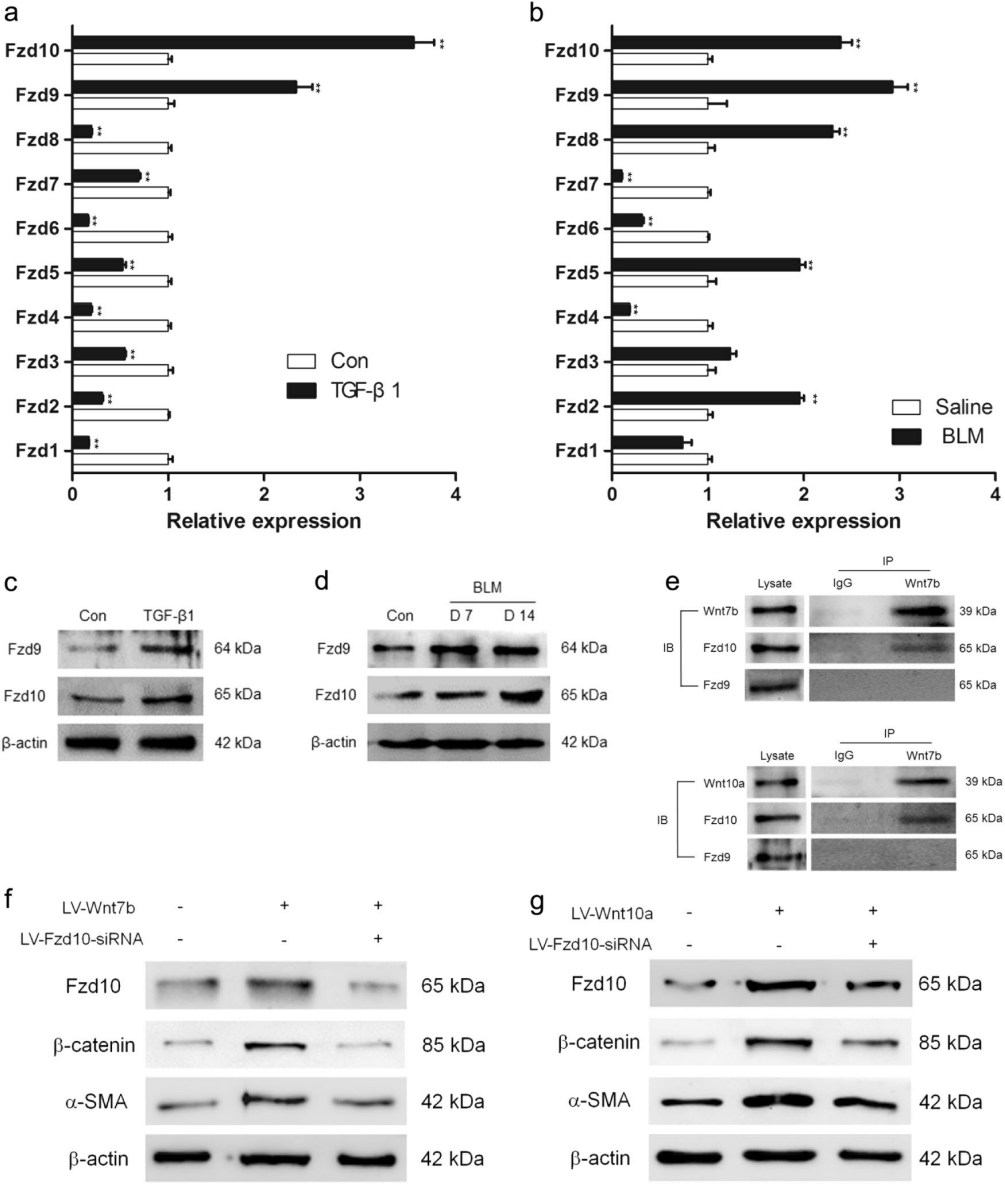
Wnt7b- and Wnt10a-induced activation of canonical Wnt signaling were mediated through Fzd10. **a**, **b** The mRNA expression of Fzd in TGF-β1-treated lung-resident mesenchymal stem cells (LR-MSCs) and mouse fibrotic lung tissues were measured by quantitative PCR. ** *P* < 0.05 vs. Con or Saline. **c**, **d** The protein levels of Fzd9 and Fzd10 were determined by western blot. **e** Immunoblotting of Fzd10 and Wnt7b in the Wnt7b immunoprecipitate and immunoblotting of Fzd10 and Wnt10a in the Wnt10a immunoprecipitate from mouse fibrotic lung tissues were analyzed. **f**, **g** LR-MSCs were transfected with either 5 × 10^7^ TU/ml of LV-Wnt7b or LV-Wnt10a. Some of the cells were co-transfected with LV-Fzd10-siRNA. The expression of Fzd10, α-SMA and β-catenin was examined by western blot

**Fig. 9:**
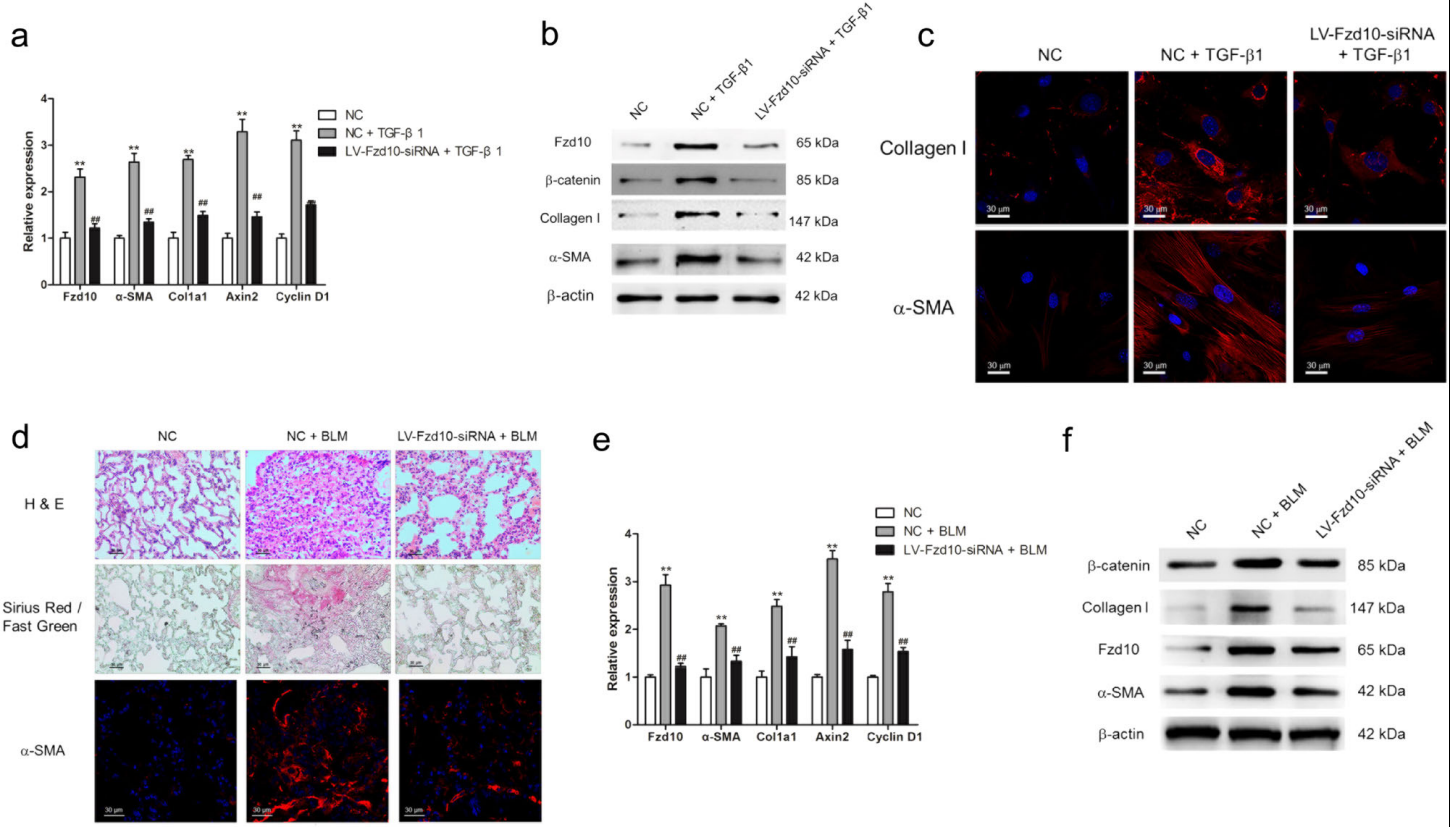
Silence of Fzd10 could suppress myofibroblast differentiation of lung-resident mesenchymal stem cells (LR-MSCs) and pulmonary fibrogenesis. **a**–**c** LR-MSCs were transfected with LV-Fzd10-siRNA followed with the treatment of TGF-β1 for 7 days. The mRNA levels of Fzd9, α-SMA, Col1a1, Axin2 and Cyclin D1 were measured by quantitative PCR (**a**). The protein levels of Fzd10, β-catenin, Collagen I and α-SMA were determined by western blot (**b**). The expression of Collagen and α-SMA were further examined by immunofluorescence staining (**c**). **d**–**f** Mice were intratracheally injected with 5 × 10^8^ TU/ml LV-Fzd10-siRNA or negative control (NC) 7 days after administration of bleomycin (BLM). Mice were killed at day 14 after BLM instillation. Pulmonary fibrosis was determined by hematoxylin–eosin (H&E) staining and collagen was revealed by Sirius Red/Fast Green staining. The expression of α-SMA was further confirmed by immunofluorescence staining (**d**). The mRNA levels of Fzd10, α-SMA, Col1a1, Axin2 and Cyclin D1 were determined by quantitative PCR (**e**). The protein levels of Fzd10, β-catenin, Collagen I and α-SMA were measured by western blot (**f**). **P < 0.01 vs. NC, ^##^*P* < 0.01 vs. NC+BLM

As the activation of canonical Wnt signaling was mediated through the interaction between Wnt ligand and Fzd receptor, the mRNA level of Fzd family was further measured in our study. As shown in Fig. 8a, b, Fzd9 and Fzd10 were dramatically increased in myofibroblast-differentiated LR-MSCs and fibrotic lungs. The protein expression of Fzd9 and Fzd10 were upregulated in vitro and in vivo as well (Fig. 8c, d). Therefore, we further investigated whether Wnt7b/10a could interact with Fzd9 or Fzd10. As shown in Fig. 8e, Wnt7b/10a could bind with Fzd10 but not Fzd9. In addition, inhibiting the expression of Fzd10 could effectively suppress the level of β-catenin and α-SMA which was increased by elevating the expression of Wnt7b or Wnt10a (Fig. 8f, g). These results demonstrated that Fzd10 mediated Wnt7b or Wnt10a-induced activation of canonical Wnt signaling and myofibroblast differentiation of LR-MSCs. We also proved that inhibition of Fzd10 could attenuate TGF-β1-induced Wnt/β-catenin activation and myofibroblast differentiation of LR-MSCs (Fig. 9a–c).

### Mice are protected from BLM-induced pulmonary fibrosis through suppressing Fzd10

We further analyzed the effect of Fzd10 inhibition on BLM-induced pulmonary fibrosis. As shown in Fig. 9d, pulmonary fibrotic lesion and collagen deposition were greatly reduced after LV-Fzd10-siRNA administration (Fig. 9d). Administration of LV-Fzd10-siRNA profoundly suppressed the expression of Fzd10, Col1a1 and α-SMA as demonstrated by quantitative PCR (q-PCR) and western blot (Fig. 9e, f). In addition, silencing Fzd10 could attenuate the activation of Wnt/β-catenin signaling through impairing the expression of β-catenin and the mRNA levels of Wnt signaling target genes, including Axin2 and Cyclin D1 (Fig. 9e, f). Immunofluorescence staining also revealed that LV-Fzd10-siRNA retarded myofibroblast activation in BLM-induced pulmonary fibrosis (Fig. 9d). These results indicated that inhibition of Fzd10 could repress the activation of canonical Wnt signaling and reduce pulmonary fibrosis after injury.

## Discussion

IPF is a fatal lung disease characterized by the progressive and irreversible destruction of lung architecture. It was well accepted that repetitive injury, in the presence or absence of local inflammation, contributed to deregulated wound repair and ECM accumulation^32^. In our previous research, we found that activating Wnt/β-catenin signaling could induce LR-MSCs to differentiate into myofibroblasts which were the principal components of myofibroblast foci^33^. Wnt signaling plays an essential role in the development and maintenance of multiple organ systems, such as the brain, intestines, skin, hematopoietic and lung^34–36^. Recent studies have demonstrated that Wnt signaling was aberrantly activated in IPF, indicating a role for this pathway in the pathogenesis of human fibrosis^7,8^. It was well known that Wnt/β-catenin signaling was mainly activated by Wnt proteins. It was ever reported that a number of Wnt genes, including Wnt2, Wnt5a, Wnt7b, Wnt11 and Wnt13, were expressed in both developing and adult lung^34^. Thus, 19 Wnt genes were measured in mouse fibrotic lung tissues and myofibroblast-differentiated LR-MSCs. In fact, most Wnt genes were upregulated during the entire experimental period, which suggested that Wnt family was positively responsive to injurious stimuli both in vivo and in vitro. Among these upregulated Wnt family members, Wnt7b and Wnt10a were ever reported to be highly expressed in the lung tissues from IPF patients as well^15,29^.

These findings are of special interest, as the prognosis for IPF is poor for unresponsiveness to currently available therapies. While historically the inflammatory processes were thought to trigger and facilitate the development of IPF^1^, this view was questioned for the ineffectiveness of anti-inflammatory therapy in IPF^2,37^. Recently, a major key pathophysiological event in IPF that was under discussion was repetitive injury without appropriate repair and subsequent myofibroblast differentiation of MSCs^38^. Our observations in vivo proved that LR-MSCs (Sca-1^+^) could differentiate into myofibroblasts (α-SMA^+^) in BLM-induced pulmonary fibrosis (Fig. 1c). To confirm the role that Wnt7b and Wnt10a play in the myofibroblast differentiation of LR-MSCs, overexpression of Wnt7b and Wnt10a could activate canonical Wnt signaling and promote LR-MSCs to differentiate into myofibroblasts (Fig. 7). Canonical Wnt signaling activation was initially mediated through interactions between Wnt ligands and Fzd receptors^35^. As the expressions of Fzd9 and Fzd10 were evidently upregulated in pulmonary fibrogenesis, it is plausible to assume that Wnt7b/10a and Fzd9/10 could be the potential targets in IPF therapy. One of the striking observation in this study is demonstrating the interaction of Wnt7b/10a and Fzd10 (Fig. 8e). As a receptor for the Wnt pathway, Fzd10 was expressed at high level in several cancers, including gastric cancers and colorectal cancers^39,40^. Fzd10 could activate canonical Wnt/β-catenin signaling in colorectal cancer cells^41^. In our work, we demonstrated that Wnt7b/10a-induced canonical Wnt signaling cascade could be effectively suppressed by silencing Fzd10 (Fig. 8f, g). In addition, inhibition of Fzd10 could impair TGF-β1-induced myofibroblast differentiation of LR-MSCs and alleviate BLM-induced pulmonary fibrosis (Fig. 9). This finding in the report provides a clear link between Wnt7b/10a, Fzd10 and pulmonary fibrosis, indicating that controlling the synthesis of Wnt proteins could avert the aberrant activation of Wnt signaling and retard the progression of pulmonary fibrosis.

It was reported that activating hedgehog signaling could result in nuclear translocation of Gli which could bind with the promoter area of Wnt genes^21,22^. In the current study, we identified whether there were potential links among hedgehog signaling, Wnt signaling, and myofibroblast differentiation of LR-MSCs that would eventually lead to pulmonary fibrosis. Hedgehog/Gli system was expressed in the adult lung but was activated in the IPF lung and repressed in the normal lung^42^. Nuclear localization of GLI1 and GLI2, the final and bona fide hallmark of hedgehog signaling activation, was detected only in IPF lungs, particularly in fibroblastic foci^42^. Inhibiting the activity of Gli in the nucleus with GANT61 completely reverted the differentiation of myofibroblasts from normal or IPF lung tissues^42^. In our research, we found that Gli1 was increased much more significantly than the expression of Gli2 in the development of pulmonary fibrosis (Fig. 4). Findings in mouse mutants suggested that Gli2 was important for the activator function in response to hedgehog signaling, while Gli1 primarily functions as the amplifier of transcriptional response^43–47^. In mammals, Gli1-KO mice develop normally^47,48^, whereas Gli2-KO mice die at birth with several skeletal and neural defects^49,50^. Given the specific inhibitor of Gli1 (GANT58) in LR-MSCs, we demonstrated that GANT58 could suppress LR-MSCs to differentiate into myofibroblasts (Fig. 5f–h). Furthermore, in BLM-induced lung fibrosis, GANT58 decreased fibrosis severity (Fig. 5a–e). Interestingly, the treatment of GANT58 in vitro or in vivo resulted in a reduction of most Wnt genes (e.g., Wnt7b and Wnt10a) that were upregulated in pulmonary fibrogenesis (Fig. 6), suggesting the potential regulation mechanism between Gli1 and Wnts. Gli1 could induce transcription of at least three Wnt family members, Wnt2b, Wnt4 and Wnt7b^51^. As transcription factors, activated Gli could bind to the GACCACCCA-like motif for the transcriptional regulation of hedgehog target genes^22^. Our results indicated that, in LR-MSCs, Gli1 played a critical role in regulating the expression of Wnt7b and Wnt10a. We further demonstrated that Wnt7b and Wnt10a were direct targets of Gli1 which could bind to their promoter areas (Fig. 6).

**Fig. 10:**
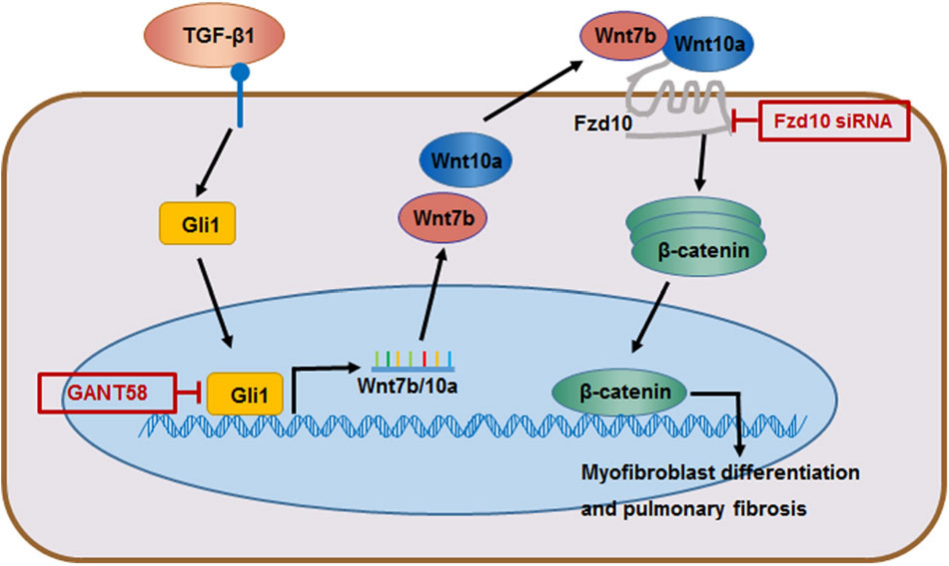
Schematic illustration of the functions of Wnt/β-catenin and hedgehog signaling in modulation of myofibroblast differentiation of lung-resident mesenchymal stem cells and pulmonary fibrosis. In the process of pulmonary fibrosis, TGF-β1 could activate Hedgehog pathway via inducing Gli1 translocate to nucleus, which promotes the transcription and translation of Wnt7b and Wnt10a through binding with their promoter areas. Furthermore, Wnt7b and Wnt10a can directly bind with Fzd10 receptor and induce the activation of Wnt/β-catenin signaling, leading to myofibroblast differentiation and pulmonary fibrosis. Oppositely, inhibition of Gli1 or knockdown Fzd10 can suppress myofibroblast differentiation and pulmonary fibrosis

Taken together, we proposed a model in which hedgehog signaling was activated in myofibroblast differentiation of LR-MSCs with the stimulation of TGF-β1. Then, Gli1 binded to the promoter area of Wnt7b/10a to enhance the transcription of the two genes. The excessive Wnt7b/10a were secreted and further activated Wnt/β-catenin signaling through interacting with their receptor Fzd10 (Fig. 10).

## Methods

### Ethics statement

The animal experiments were performed according to the Guide for the Care and Use of Laboratory Animals (The Ministry of Science and Technology of China, 2006) and all experimental protocols were approved under the animal protocol number SYXK (Su) 2009-0017 by the Animal Care and Use Committee of Nanjing University.

### Antibodies

Mouse monoclonal antibody against mouse β-actin (ab8277), rabbit polyclonal antibody against mouse Gli1 (ab49314), rabbit monoclonal antibody against mouse β-catenin (ab32572), rabbit polyclonal antibody against mouse α-SMA (ab5694), rabbit monoclonal antibody against mouse Vimentin (ab92547), rabbit polyclonal antibody against mouse Collagen I (ab34710), rabbit polyclonal antibody against mouse Fzd9 (ab195718) and rabbit polyclonal antibody against mouse Fzd10 (ab137491) were purchased from Abcam (Cambridge, MA). Mouse monoclonal antibody against mouse Wnt10a was purchased from Santa Cruz (Beverly, MA). Rabbit monoclonal antibody against mouse Wnt7b was purchased from Bioss (Beijing, China).

### Cell culture and transfection

Isolation of LR-MSCs was performed as previously reported^44^. Freshly isolated LR-MSCs were cultured at a concentration higher than 105 cells/ml with Dulbecco's modified Eagle's medium (Grand Island, NY, Gibco) containing 15% fetal bovine serum (Gibco), 4% l-glutamine, 1% nonessential amino acids and 1% penicillin and streptomycin, and maintained in a humidified atmosphere of 95% air and 5% CO_2_ at 37 °C. The cells were passaged 1:2 using 0.25% trypsin when they reached 70–90% confluence. Transfections were performed with lentiviral protocols provided by GENECHEM (Shanghai, China). The LV-Gli1, LV-Wnt7b, LV-Wnt10a and LV-Fzd10-siRNA vectors were synthesized by GENECHEM. The transfection efficiency was measured by quantitative real-time PCR (qRT-PCR) and western blot.

### Induction and treatment of pulmonary fibrosis

All animal procedures were conducted in accordance with humane animal care standards approved by the Nanjing University Ethics Committee (Nanjing, China) and maintained under specific pathogen-free conditions. The animals were acclimated to the environment for 1 week prior to treatment. The mice (*n* = 6) were administered with BLM (Nippon Kayaku, Tokyo, Japan) intratracheally at a dose of 5 mg/kg dissolved in a total of 50 μl sterile saline. The control group was similarly treated with 50 μl of sterile saline.

To investigate the role of hedgehog signaling in pulmonary fibrosis, mice were treated with GANT58 (MedChem Express, San Diego, CA), which was known to inhibit hedgehog signaling by directly blocking the binding of Gli1 to their DNA targets^52,53^. GANT58 (25 mg/kg) or vehicle was injected intraperitoneally every day from day 7 to day 13. Mice were killed at day 14, and lung tissues were collected for further analysis.

In order to investigate the effect of Fzd10 inhibition on pulmonary fibrogenesis, the mice were administered with BLM intratracheally at a dose of 5 mg/kg dissolved in a total of 50 μl sterile saline. After 3 days, BLM-treated mice were intratracheally injected with LV-Fzd10-siRNA or NC at a dose of 4 × 10^8^ TU/ml diluted by sterile saline. The mice were killed for lung collection at day 14 after BLM administration.

### Sodium dodecyl sulfate–polyacrylamide gel electrophoresis and immunoblotting

Briefly, whole cell or tissue lysates were separated on 12% sodium dodecyl sulfate–polyacrylamide gels and transferred to a polyvinylidene fluoride membrane (Roche, Germany) by standard procedures. Membranes were blocked by incubation for 1 h with 5% non-fat milk in phosphate-buffered saline (PBS) containing 0.5% Tween-20 (PBST) and blotted with specific antibodies at 4 °C for 12 h. After three washes in PBST, the membranes were incubated with the secondary antibody at 37 °C for 1 h. Immunoreactive protein bands were detected using an Odyssey Scanning System (LI-COR, Lincoln, NE).

### Quantitative real-time PCR

For analysis of mRNA, HiScript 1st strand cDNA Synthesis Kit (Vazyme Biotech Co., Nanjing, China) was used for RT-PCR reaction. Gene expression was quantified by SYBR Green Q-PCR Kit (Roche, Germany) using the ABI Prim 7300 Sequence Detection System (Applied Biosystems, Foster city, CA). Specific primers for mRNAs are listed in Supplementary Table 1. The Ct values were analyzed using the ΔΔCt method and relative changes of mRNA levels were obtained by normalization to glyceraldehyde-3-phosphate dehydrogenase (GAPDH) relative to the control.

### Histopathology

The mouse lungs were inflated with a neutral buffered formalin solution overnight and embedded in paraffin before sectioning into 5 μm-thick slices. The sections were stained with hematoxylin–eosin for structure observation, or used for detection of collagen deposition by Sirius Red/Fast Green Collagen Staining Kit (Chondrex, MA).

### Immunohistochemistry

The 5 μm-thick paraffin-embedded sections were deparaffinized with xylene (twice for 5 min each) before being rehydrated in water using an ethanol gradient. After washing with water, antigen retrieval was performed in a steamer using citrate buffer (pH 6.0, DAKO) for 20 min, and the samples were then cooled to room temperature. The sections were then washed with PBST, incubated with 3% H_2_O_2_ for 10 min and blocked with the avidin/biotin blocker and the serum-free blocking reagent. The sections were subsequently incubated with rabbit anti-Gli1, mouse anti-Wnt7b or mouse anti-Wnt10a overnight at 4 °C.The DAB or AEC Substrate System (DAKO) was used to reveal the immunohistochemical staining.

### Immunofluorescent staining

The immunofluorescence analysis was performed as previously described^28^. Rabbit anti-Collagen I and mouse anti-α-SMA were employed as the primary antibodies. Alexa Fluor 594-conjugated goat anti-mouse/rabbit IgG (Invitrogen) was used as the secondary antibody. Nuclei were stained with 1 μg/ml 4',6-diamidino-2-phenylindole (DAPI; Sigma). The images were captured using a confocal fluorescence microscope (Olympus, Tokyo, Japan).

### Chromatin immunoprecipitation

Cells for ChIP were cultured in 10 × 10 cm dishes. The ChIP assay was performed by following the instructions of Pierce Agarose ChIP Kit (Thermo Scientific). LR-MSCs were subjected to cross-linking with 1% formaldehyde. After stopping the reaction with 0.1 M glycine, the chromatin was sheared into fragments of 500–1000 bp in length and the DNA–protein complex of chromatin fragments was precipitated by anti-Gli1 (Santa Cruz) or anti-IgG antibody provided by the kit. DNA was then eluted and extracted with phenol–chloroform and subjected to PCR. Wnt7b or Wnt10a promoter-specific primers were used to amplify the Gli1 binding regions. The primers were as follows: Wnt7b promoter sense, 5′-ACGTCTAGGGGAAAGATGTCT-3′, Wnt7b promoter antisense, 5′-CTCAGGGCTTGGTGGTCAC-3′; Wnt10a promoter sense, 5′-GCTGCATATCCCAGGCTT-3′, Wnt10a promoter antisense, 5′-GGTGCCCACAGGAACAGG-3′.

### Co-immunoprecipitation (Co-IP) and protein analysis

Co-IP was performed using the Thermo Scientific Pierce Co-IP kit following the manufacture’s protocol. Briefly, the Wnt7b or Wnt10a antibody (Santa Cruz) was first immobilized for 2 h using AminoLink Plus Coupling Resin. The resin was then washed and incubated with the lysate of lung tissues overnight. A negative control that was provided with the IP kit to assess nonspecific binding received the same treatment as the Co-IP samples. Samples were further analyzed by western blot^54^.

### Statistical analysis

The data are presented as mean values ± SD. Differences were analyzed for significance (*P* < 0.05) by one-way analysis of variance using SPASS for windows version 11.0 (SPASS, Chicago, IL).

## Electronic supplementary material

Supplementary Table 1

## References

[1] Gross TJ, Hunninghake GW (2001). Idiopathic pulmonary fibrosis. N. Engl. J. Med..

[2] Selman M, King TE, Pardo A (2001). Idiopathic pulmonary fibrosis: prevailing and evolving hypotheses about its pathogenesis and implications for therapy. Ann. Intern. Med..

[3] Selman M (2006). Gene expression profiles distinguish idiopathic pulmonary fibrosis from hypersensitivity pneumonitis. Am. J. Respir. Crit. Care Med..

[4] Thannickal VJ, Toews GB, White ES, Lynch JP, Martinez FJ (2004). Mechanisms of pulmonary fibrosis. Annu. Rev. Med..

[5] King TE (2001). Idiopathic pulmonary fibrosis: relationship between histopathologic features and mortality. Am. J. Respir. Crit. Care. Med..

[6] Nicholson AG (2002). The relationship between individual histologic features and disease progression in idiopathic pulmonary fibrosis. Am. J. Respir. Crit. Care Med..

[7] Chilosi M (2003). Aberrant Wnt/beta-catenin pathway activation in idiopathic pulmonary fibrosis. Am. J. Pathol..

[8] Konigshoff M (2008). Functional Wnt signaling is increased in idiopathic pulmonary fibrosis. PLoS One.

[9] Logan CY, Nusse R (2004). The Wnt signaling pathway in development and disease. Annu. Rev. Cell Dev. Biol..

[10] van Amerongen R, Nusse R (2009). Towards an integrated view of Wnt signaling in development. Development.

[11] Huang H, He X (2008). Wnt/beta-catenin signaling: new (and old) players and new insights. Curr. Opin. Cell Biol..

[12] Macdonald BT, Semenov MV, He X (2007). SnapShot: Wnt/beta-catenin signaling. Cell.

[13] Nusse R (2005). Wnt signaling in disease and in development. Cell Res..

[14] Winn RA (2005). Restoration of Wnt-7a expression reverses non-small cell lung cancer cellular transformation through frizzled-9-mediated growth inhibition and promotion of cell differentiation. J. Biol. Chem..

[15] Meuten T (2012). WNT7B in fibroblastic foci of idiopathic pulmonary fibrosis. Respir. Res..

[16] He W (2009). Wnt/beta-catenin signaling promotes renal interstitial fibrosis. J. Am. Soc. Nephrol..

[17] Yu F, Fan X, Chen B, Dong P, Zheng J (2016). Activation of hepatic stellate cells is inhibited by microRNA-378a-3p via Wnt10a. Cell. Physiol. Biochem..

[18] Ortiz LA (2003). Mesenchymal stem cell engraftment in lung is enhanced in response to bleomycin exposure and ameliorates its fibrotic effects. Proc. Natl. Acad. Sci. USA.

[19] Spees JL (2007). Engraftment of bone marrow progenitor cells in a rat model of asbestos-induced pulmonary fibrosis. Am. J. Respir. Crit. Care Med..

[20] Salazar KD, Lankford SM, Brody AR (2009). Mesenchymal stem cells produce Wnt isoforms and TGF-beta1 that mediate proliferation and procollagen expression by lung fibroblasts. Am. J. Physiol. Lung Cell Mol. Physiol..

[21] Katoh M, Katoh M (2009). Transcriptional mechanisms of WNT5A based on NF-kappaB, Hedgehog, TGFbeta, and Notch signaling cascades. Int. J. Mol. Med..

[22] Katoh M, Katoh M (2009). Transcriptional regulation of WNT2B based on the balance of Hedgehog, Notch, BMP and WNT signals. Int. J. Oncol..

[23] Yang SH (2008). Pathological responses to oncogenic Hedgehog signaling in skin are dependent on canonical Wnt/beta3-catenin signaling. Nat. Genet..

[24] Bolanos AL (2012). Role of Sonic Hedgehog in idiopathic pulmonary fibrosis. Am. J. Physiol. Lung Cell Mol. Physiol..

[25] Chen MH (2009). Cilium-independent regulation of Gli protein function by Sufu in Hedgehog signaling is evolutionarily conserved. Genes Dev..

[26] Kim J, Kato M, Beachy PA (2009). Gli2 trafficking links Hedgehog-dependent activation of Smoothened in the primary cilium to transcriptional activation in the nucleus. Proc. Natl. Acad. Sci. USA.

[27] Wilson CW, Chuang PT (2010). Mechanism and evolution of cytosolic Hedgehog signal transduction. Development.

[28] Chen X (2017). The role of miR-497-5p in myofibroblast differentiation of LR-MSCs and pulmonary fibrogenesis. Sci. Rep..

[29] Oda K (2016). Profibrotic role of WNT10A via TGF-beta signaling in idiopathic pulmonary fibrosis. Respir. Res..

[30] Radtke F, Clevers H, Riccio O (2006). From gut homeostasis to cancer. Curr. Mol. Med..

[31] Katoh M (2007). Networking of WNT, FGF, Notch, BMP, and Hedgehog signaling pathways during carcinogenesis. Stem. Cell Rev..

[32] Selman M, Pardo A, Kaminski N (2008). Idiopathic pulmonary fibrosis: aberrant recapitulation of developmental programs?. PLoS Med..

[33] Chen X (2016). Inhibition of Wnt/beta-catenin signaling suppresses bleomycin-induced pulmonary fibrosis by attenuating the expression of TGF-beta1 and FGF-2. Exp. Mol. Pathol..

[34] Morrisey EE (2003). Wnt signaling and pulmonary fibrosis. Am. J. Pathol..

[35] Moon RT, Kohn AD, De Ferrari GV, Kaykas A (2004). WNT and beta-catenin signalling: diseases and therapies. Nat. Rev. Genet..

[36] Staal FJ, Clevers HC (2005). WNT signalling and haematopoiesis: a WNT-WNT situation. Nat. Rev. Immunol..

[37] Gauldie J, Kolb M, Sime PJ (2002). A new direction in the pathogenesis of idiopathic pulmonary fibrosis?. Respir. Res..

[38] Skurikhin EG (2013). Differentiation of mesenchymal multipotent stromal cells of the lungs in pneumofibrosis. Bull. Exp. Biol. Med..

[39] Nagayama S (2009). Inverse correlation of the up-regulation of FZD10 expression and the activation of beta-catenin in synchronous colorectal tumors. Cancer Sci..

[40] Fukukawa C (2009). Activation of the non-canonical Dvl-Rac1-JNK pathway by Frizzled homologue 10 in human synovial sarcoma. Oncogene.

[41] Terasaki H, Saitoh T, Shiokawa K, Katoh M (2002). Frizzled-10, up-regulated in primary colorectal cancer, is a positive regulator of the WNT - beta-catenin - TCF signaling pathway. Int. J. Mol. Med..

[42] Cigna N (2012). The hedgehog system machinery controls transforming growth factor-beta-dependent myofibroblastic differentiation in humans: involvement in idiopathic pulmonary fibrosis. Am. J. Pathol..

[43] Robbins DJ, Fei DL, Riobo NA (2012). The Hedgehog signal transduction network. Sci. Signal..

[44] Bianco P, Riminucci M, Gronthos S, Robey PG (2001). Bone marrow stromal stem cells: nature, biology, and potential applications. Stem Cells.

[45] Matise MP, Epstein DJ, Park HL, Platt KA, Joyner AL (1998). Gli2 is required for induction of floor plate and adjacent cells, but not most ventral neurons in the mouse central nervous system. Development.

[46] Litingtung Y, Chiang C (2000). Specification of ventral neuron types is mediated by an antagonistic interaction between Shh and Gli3. Nat. Neurosci..

[47] Park HL (2000). Mouse Gli1 mutants are viable but have defects in SHH signaling in combination with a Gli2 mutation. Development.

[48] Bai CB, Auerbach W, Lee JS, Stephen D, Joyner AL (2002). Gli2, but not Gli1, is required for initial Shh signaling and ectopic activation of the Shh pathway. Development.

[49] Ding Q (1998). Diminished Sonic hedgehog signaling and lack of floor plate differentiation in Gli2 mutant mice. Development.

[50] Mo R (1997). Specific and redundant functions of Gli2 and Gli3 zinc finger genes in skeletal patterning and development. Development.

[51] Moshai EF (2014). Targeting the hedgehog-glioma-associated oncogene homolog pathway inhibits bleomycin-induced lung fibrosis in mice. Am. J. Respir. Cell Mol. Biol..

[52] Lauth M, Bergstrom A, Shimokawa T, Toftgard R (2007). Inhibition of GLI-mediated transcription and tumor cell growth by small-molecule antagonists. Proc. Natl. Acad. Sci. USA.

[53] Lan X (2017). Hedgehog pathway plays a vital role in HIV-induced epithelial-mesenchymal transition of podocyte. Exp. Cell Res..

[54] Adebiyi A, Narayanan D, Jaggar JH (2011). Caveolin-1 assembles type 1 inositol 1,4,5-trisphosphate receptors and canonical transient receptor potential 3 channels into a functional signaling complex in arterial smooth muscle cells. J. Biol. Chem..

