# Supplementary material for: The hedgehog and Wnt/β-catenin system machinery mediate myofibroblast differentiation of LR-MSCs in pulmonary fibrogenesis
Source: Cell Death Dis. 2018 May 29;9(6):639. doi: 10.1038/s41419-018-0692-9 (PMC5974360; doi:10.1038/s41419-018-0692-9)
Supplement: Supplementary file 1 — Supplementary Table 1 [file 41419_2018_692_MOESM1_ESM.docx]

**Supplementary Table 1**

| Mouse Gene names | Sense | Antisense |
| --- | --- | --- |
| α-SMA | CCCAGATTATGTTTGAGACCTTC | ATCTCCAGAGTCCAGCACAATAC |
| Col1a1 | CTTCTGGTCCTCGTGGTCTCCCT | AAGCCTCGGTGTCCCTTCATTCC |
| Axin2 | GGGAGCCTAAAGGTCTTATGTGG | TCAGTGCGTCGCTGGATAACTCG |
| Cyclin D1 | TGTGAGGAGCAGAAGTGCGAAGA | GGCCGGATAGAGTTGTCAGTGTAG |
| Wnt1 | TGCGGCCCGCCTCCAGACTTATT | TGCGGTTCTGACGACTGTGGTTGCTGT |
| Wnt2 | GAGATGCTGTATGGGACTATGTAATGG | CCTGTCTGGAGGCAGTCTGAACC |
| Wnt2b | CGCACCTTCCTCTACCCTCAATC | ACTCAGCCTCCTAAATCCATCCC |
| Wnt3 | TTTGTCCTTTCCCGCTTCTGTCT | ATCCGTTCTGCTCAAGTGTCCTC |
| Wnt3a | GTATGACAGTGCCTCGGAGATGG | GTTAGGTTCGCAGAAGTTGGGTG |
| Wnt4 | AAACAGTCTCCCACCACCTACCC | AGCCAGAGGCATAAACAAAGTCC |
| Wnt5a | TGTGCTGTGGGCGTGGCTATGAC | TCCTGGGAGTGGGACTGGGTGAA |
| Wnt5b | CCAGGGCATTGGGATGGGTTGAG | CCACCAGGAGTTGGCGTCAGTCA |
| Wnt6 | CTCGGACTGCTGCTGCTGCTCTT | GCGTCGGAAACGGAACTGGAACT |
| Wnt7a | TGGCTGCTCTGCCGACATCCGCTAC | TCTTCCGACCCGCCTCGTTATTG |
| Wnt7b | GGAGAAGCAAGGCTACTACAACCAGG | GCATCCACAAAGCGACGAGAAAA |
| Wnt8a | TGCCCACATAAATACCAAGAAAGG | CACTGGCGGTGTAGGTCAGATAG |
| Wnt8b | AAGAGGATGAGAACAGGCGAGTC | CCAGAAATAGTAGGGAAATGAGAAGA |
| Wnt9a | GACTGCTTTCCTCTACGCCATCTC | TCCACTCGGGCTCGCAAATCCTT |
| Wnt9b | CCGTTGATTATTTAGGGAGTGTCTGA | GCATAGTTTATTGGTTTGTTGGCTTTC |
| Wnt10a | CCGAGTGGGTCAGCGTCTGCAAGT | CCTACAGAGGTGAGCGGAGGAATG |
| Wnt10b | TGAAATGCACTGTACTGTTAGCGTCTT | CACAATGCCTGCTATTATCCTTCG |
| Wnt11 | CCGTTTCCCTGTATGTGATCTGTTG | TGGCTGTGCTGTAAGAATGTCCCT |
| Wnt16 | GCTACTTTCTCCGCCTTATCTTGC | GCCACTCCTCCTCTGTCTCCTCT |
| Gli1 | CCACCACCCTACCTCTGTCTATTCG | AGTTCCAACCTCTGGCTCCTCCT |
| Gli2 | TTTACCCGCTCCTATTTCTCCAGT | CCTTCAACCTTCCGCTCAACCAC |
| Gli3 | ATCCACCCTGCTCCAACATTTCC | TGCTTTCATCTTTGTCCGCTTCC |
| Fzd1 | AGTATGGGCTCTGCCAGTTGACC | GGAAGAAAGTAGGTTGCCAGACATCA |
| Fzd2 | CCTCAAGGTGCCGTCCTATCTCAG | CGAAATCGCTGCATGTCCACTAAA |
| Fzd3 | CACAGTAGTTCTCATCGGCTCAA | GCACTGGTTCCATCCTCCTCAAT |
| Fzd4 | ACCCAACTCAGCATCAGAAGACC | AATCCCACCCAGTAGGAGCAAAC |
| Fzd5 | CCTAATCATCCCTCCAGCCCTATC | GCCATCGTCTCCTTCTTCCCTTT |
| Fzd6 | TGCTCGCTATGAATAAGGTTGAA | ACAGAGGCAGAAGGACGAAGTAG |
| Fzd7 | CAAGAGGAATGATAGCGAAGGAAG | ACAGTCAGTGTCAAAGGTAGGGTAAAG |
| Fzd8 | TCCGAATCCGTTCAGTCATCAAG | AGGTCCCGAAGGCATGGGCAGTT |
| Fzd9 | AAACTATCGTGGTCCTGACTCTGCG | GCTTCTCCGTATTGGTGCCTCCC |
| Fzd10 | AGAAGCTCATGGTACGCATAGGG | GGAGGTGGTCATCAGGCAGTCAG |
| GAPDH | AGGTCGGTGTGAACGGATTTG | TGTAGACCATGTAGTTGAGGTCA |
